# Predicting mortality in critically ill patients with diabetes using machine learning and clinical notes

**DOI:** 10.1186/s12911-020-01318-4

**Published:** 2020-12-30

**Authors:** Jiancheng Ye, Liang Yao, Jiahong Shen, Rethavathi Janarthanam, Yuan Luo

**Affiliations:** 1 Feinberg School of Medicine, Northwestern University, Chicago, IL USA; 2 Tencent, Shenzhen, China; 3 Dept. of Materials Science and Engineering, Northwestern University, Evanston, IL USA

**Keywords:** ICU, Diabetic disease, Clinical notes, Machine learning, Natural language processing, Mortality, Word embedding, Entity embedding, Deep learning

## Abstract

**Background:**

Diabetes mellitus is a prevalent metabolic disease characterized by chronic hyperglycemia. The avalanche of healthcare data is accelerating precision and personalized medicine. Artificial intelligence and algorithm-based approaches are becoming more and more vital to support clinical decision-making. These methods are able to augment health care providers by taking away some of their routine work and enabling them to focus on critical issues. However, few studies have used predictive modeling to uncover associations between comorbidities in ICU patients and diabetes. This study aimed to use Unified Medical Language System (UMLS) resources, involving machine learning and natural language processing (NLP) approaches to predict the risk of mortality.

**Methods:**

We conducted a secondary analysis of Medical Information Mart for Intensive Care III (MIMIC-III) data. Different machine learning modeling and NLP approaches were applied. Domain knowledge in health care is built on the dictionaries created by experts who defined the clinical terminologies such as medications or clinical symptoms. This knowledge is valuable to identify information from text notes that assert a certain disease. Knowledge-guided models can automatically extract knowledge from clinical notes or biomedical literature that contains conceptual entities and relationships among these various concepts. Mortality classification was based on the combination of knowledge-guided features and rules. UMLS entity embedding and convolutional neural network (CNN) with word embeddings were applied. Concept Unique Identifiers (CUIs) with entity embeddings were utilized to build clinical text representations.

**Results:**

The best configuration of the employed machine learning models yielded a competitive AUC of 0.97. Machine learning models along with NLP of clinical notes are promising to assist health care providers to predict the risk of mortality of critically ill patients.

**Conclusion:**

UMLS resources and clinical notes are powerful and important tools to predict mortality in diabetic patients in the critical care setting. The knowledge-guided CNN model is effective (AUC = 0.97) for learning hidden features.

## Background

Diabetes mellitus is a prevalent metabolic disease characterized by chronic hyperglycemia. The rate of incidence and prevalence of patients with Diabetes mellitus type 2 among adults is increasing over time and has led to an increase in the number of patients admitted in the intensive care unit (ICU). These diabetic patients use more than 45% of resources in the ICU compared to the patients associated with other chronic diseases [1]. Additionally, it is well known that patients admitted in ICU due to diabetes are more prone to diseases and risk complication; one of these risk factors is due to the hampered immune cell response to the disease [2]. Furthermore, these risks can directly impact the survival of diabetic patients in the ICU. Only a few studies have been conducted on the mortality of diabetes mellitus patients; most of them are limited to factors associated with the increased mortality in the ICU setting [3].

The prognostic models developed previously were based on the Cox regression model and linear regression models. These models work best when the duration of diabetes is known and the data such as cohort characteristics, is a contributing factor [4]. To date, only a few studies have taken various combinations of factors into consideration to predict mortality. Anand et al. used predictive modeling, along with a combination of five key variables (type of admission, mean glucose, hemoglobin A1c, diagnoses, and age), to predict mortality, which achieved a fit with AUC values of 0.787 [3].

Meanwhile, the clinical notes that contain the patients’ medical records are considered important resources to solving critical clinical issues that are difficult to obtain from other components of the electronic health records (EHR), such as laboratory data. When processed, these notes in natural language provide detailed patient information and help with clinical reasoning and inferences [5, 6]. More recently, machine learning algorithms, natural language processing (NLP), and deep learning models have been utilized to perform text processing and classification for understanding intensive care risks. These approaches have been taking into consideration the physiological [7, 8], vital [9] and medication profiles [10].

Recently, text classification methods have been suggested to help in clinical document clustering; for example, some studies have utilized automated clinical document clustering for diagnosis identification and clinical procedures [11], identifying adverse drug effects [10], etc. Lexical features, such as bag-of-words or bag-of-concepts approach, are used by integrating medical ontologies, such as Unified Medical Language System (UMLS) Metathesaurus to embed clinical knowledge as machine computable information [12]. The state-of-art approach for text classification uses deep learning algorithms, such as neural network models with the distributed clinical text representation, and can learn complicated entity embeddings with the algorithms itself [11]. For instance, Yao et al. applied convolutional neural networks (CNN) with word embedding and UMLS entity embeddings to recognize and predict classes using trigger phrases [13]. Their work showed that combining domain knowledge and CNN models are promising for clinical text classification and outperforming obesity challenges [13]. Similarly, Hughes et al. utilized a deep learning algorithm at the sentence level for word representation with regard to medical text classification and were able to achieve a competitive model performance [14]. Domain knowledge in health care is built on the dictionaries created by experts who defined the clinical terminologies such as medications or clinical symptoms. This knowledge is valuable to identify information from text notes that assert a certain disease. Knowledge-guided models can automatically extract knowledge from clinical notes or biomedical literature that contains conceptual entities and relationships among these various concepts [15].

The avalanche of healthcare data is accelerating precision and personalized medicine. Artificial intelligence and algorithm-based approaches are becoming more and more vital to support clinical decision-making, health care delivery and health services [16–18]. These methods are able to augment health care providers by taking away some of their routine work and enabling them to focus on critical issues [19, 20]. In this study, we proposed a new method that combines machine learning and knowledge-guided feature extraction to predict mortality among patients with diabetes mellitus. Additionally, our work demonstrates that effectively applying NLP to clinical notes and extracting meaningful features can lay the foundation for building machine learning models that are predictive for mortality in critically ill patients with diabetes. From a practical point of view, our prediction model could be used to better understand and forecast the mortality risks for critically ill patients with diabetes.

## Methods

Data were extracted from Medical Information Mart for Intensive Care-III (MIMIC-III) data using SQL queries [21]. The database contains information regarding ICU admission, medications, vitals, duration of stay, ICD-9-CM diagnosis and laboratory reports. The patients with ICD-9-CM diagnosis code for diabetes mellitus (Diabetes type 1 and 2, secondary and gestational diabetes) admitted in the ICU. Pre-processing and analyses were performed using Python programming. The diabetes severity index was calculated with the points assigned for the specific ICD-9-CM codes, and the predictive model of mortality was generated with test and training sets using Python scikit-learn packages for machine learning and statistical analysis [22]. The predictive model pipeline was constructed using the clinical NLP system, clinical text classification, knowledge extraction system, the UMLS Metathesaurus, Semantic Network and learning algorithms. Multiple ICU encounters of the same patients were assigned into either a held-out test set or the training set, their information was concatenated together to form one record.

### Data processing

All data processing was conducted using the Python programming language. The variables of gender, type of diabetes and severity score were calculated for each patient. The severity score was measured by the degree of organ dysfunction using the sequential organ failure assessment (SOFA) score [23]. The six organ system subscores (i.e. respiratory, coagulation, hepatic, cardiovascular, neurologic, and renal) of SOFA were scaled from 0 (no dysfunction) to 4 (severe dysfunction). The six subscores were measured in 24-h periods for the first 72 h of stay in all patients, and the highest score achieved was used as the clinical feature for clustering [24]. Simplified Acute Physiology Score II (SAP II) [25] and Acute Physiology Score III (APS III) [26] were calculated following the standard guidelines. These clinical information are useful to validate the performance of the models. The demographic data, clinical data and the severity score were merged into a single data frame for further analysis. The entire dataset was split in the approximate ratio of 7:3 to the training and testing sets.

### Clinical word and text representation

Text classification is useful to present medical language that can be leveraged to learn the phrases that are relevant to the medical condition in the clinical notes. NLP models can extract this valuable information, in conjunction with structured data analysis, can lead to a better understanding of the diseases [27] and a more precise phenotyping of the patients [28]. The intelligent phenotyping can assist clinical decision support by improving the workflow and reviewing clinical charts, etc. The text classification was performed using phenotyping models—CNN. MetaMap [29] was applied when we identified medical concepts from clinical notes in the MIMIC-III dataset. The extracted medical concept features were from UMLS.

The UMLS Metathesaurus was used to filter clinically relevant concepts in the clinical notes [12]. To acquire UMLS concept unique identifiers (CUIs), the entity representations were used to identify and normalize lexical variants from the unstructured text content. The full clinical text was linked to CUIs in UMLS [12] via MetaMap. After entity linking, each clinical record was represented as a bag of CUIs. The UMLS CUIs were restricted within clinically relevant semantic groups and types. The neural word embedding model, word2vec, was utilized to learn word embeddings from different corpora using the continuous bag-of-words method [30].

### Predictive machine learning models

The mortality rate of the patients was the primary outcome of the predictive model and we studied prediction risks of hospital mortality. The machine learning models were used to predict which diabetic patients are most likely to die in the ICU, thus providing better treatment guidance to health care providers. All model fitting was conducted using packages from Python Scikit-learn packages. The package was used to fit the regression model that contained all the relevant variables [3] to determine which variables have the greatest impact on mortality. The GLM package was used to fit the binominal logistic regression model. In the model, 70% of the sample was used as the training set, while the remaining 30% of the sample was used for validation. The key variables for these statistical machine learning predictive models include social demographics variables, such as age, gender and race, and critical clinical variables, such as hospital length of stay, SOFA scores, SAPS II, and APS III. All the feature variables are shown in Table 1; they were used in bivariate analyses to correlate with the prediction of mortality risk. The p-values less than 0.05 were considered significant for all the variables for multivariate analysis.

**Table 1:** Characteristics of diabetic patients in ICU

	Mortality (n = 1164)	Survival (n = 8790)
Gender, No. (%)		
Male	658 (56.53)	5118 (58.23)
Female	506 (43.47)	3672 (41.77)
Age, median (IQR), y	73.51 (63.16–80.36)	66.99 (57.35–76.23)
Race/ethnicity, No. (%)		
White non-hispanic	762 (65.46)	5879 (66.88)
Black non-hispanic	117 (10.05)	1045 (11.89)
Hispanic	39 (3.35)	382 (4.35)
Asian	23 (1.98)	199 (2.26)
Other	223 (19.16)	1285 (14.62)
Hospital LOS, median (IQR), d	6.27 (2.07–13.76)	7.29 (4.56–12.10)
Max subscores in the first 72 h, median (IQR)		
Respiration	2 (0–3)	0 (0–2)
Coagulation	0 (0–1)	0 (0–1)
Hepatic	0 (0–1)	0 (0–0)
Cardiovascular	1 (1–4)	1 (1–1)
Neurologic	3 (2–4)	2 (0–4)
Renal	1 (0–2)	0 (0–1)
SAPS II, median (IQR)	50 (39–61)	33 (26–42)
APS III, median (IQR)	63.5 (48–81)	40 (31–53)
SOFA_mean in the first 7 days, median (IQR)	6.67 (4.33–9.4)	3 (1.71–4.67)
SOFA_median in the first 7 days, median (IQR)	7 (4–10)	3 (1.5–5)

*SOFA* Sequential Organ Failure Assessment, *IQR* interquartile range, *LOS* length of stay, *MODS* multiple organ dysfunction syndrome, *SAPS II* Simplified Acute Physiology Score II, *APS-III* Acute Physiology Score III (APS) III

Following the logistic regression model, we built a random forest model to predict mortality risk using the RandomForestClassifier package with sklearn. The variables extracted from the MIMIC-III database were used in the analysis. The model was initially trained with a single decision tree, and the depth was further increased until train and test sets began to diverge. Probability estimates were used to plot the Receiver operating curve (ROC) curve. The ROC curves were generated by altering the thresholds of the machine learning models. The performance of all the employed models were compared by area under the curve (AUC) measures. Furthermore, we evaluated more machine learning models on this diabetic cohort. (Table 2).

**Table 2:** Performance of machine learning models

	AUC	PPV	TPR	F1 score
Logistic regression	0.82	0.63	0.25	0.35
Random forest	0.86	0.81	0.34	0.48
AdaBoost	0.84	0.68	0.32	0.44
Gradient boosting	0.83	0.55	0.39	0.46
XGBoost	0.87	0.77	0.37	0.50
ANN	0.86	0.77	0.34	0.47
Majority voting	0.87	0.82	0.33	0.47

*AUC* area under the curve, *PPV* positive predictive value, *TPR* true positive rate

### Knowledge-guided convolutional neural networks

To apply the Knowledge-guided CNN to clinical notes, we first identified trigger phrases using the rule that was developed to tackle semantic classification tasks [31], which were then utilized to predict classes. The trigger phrases are the name of diseases and their alternative synonyms. Next, a CNN on the trigger phrases with word embeddings and UMLS CUIs were trained. We used the Knowledge-guided CNN to combine CUI features and word features. It employed CUIs embeddings of clinical notes and pre-trained word embeddings as the input. The input layer contained word embeddings and entity embeddings of selected CUIs in each clinical record. Max pooling was utilized to select the most prominent features that have the highest values in the convolutional feature map. After that, the max pooling results of entity and word embeddings were concatenated. We adopted the same parameter settings for Knowledge-guided CNN from a previous study [13]; the convolution kernel size was 5, the number of convolution filters was 256, the dimension of hidden layer in the fully connected layer was 128, dropout keep probability was 0.8, the number of learning epochs was 30, batch size was 64, learning rate was 0.001. To address imbalance, we experimented with random under-sampling with the training class ratio as 1:3. Under-sampling was employed to improve the classifiers to a reasonable range,;some observations in the majority class were removed [32].

### Results

Table 1 presents characteristics of diabetic patients in the ICU. There were 9954 patients in the MIMIC-III with different types of diabetes (Diabetes type 1 and 2, secondary and gestational diabetes). 1164 (11.69%) of them died during the hospital course, while 8790 (88.31%) survived. Those surviving patients had a longer hospital stay (median = 7.26). We also measured the degree of organ dysfunction using the sequential organ failure assessment (SOFA) score [23] in patients admitted to the ICU. The six subscores were measured in 24-h periods for the first 72 h of stay in all patients, and the highest score achieved was used as the clinical feature for clustering. The 72-h time window was chosen as a proxy for the early phase of critical illness and because a large portion of organ dysfunctions tend to peak within the first days after ICU admission [33]. We also included the Simplified Acute Physiology Score (SAPS) II and Acute Physiology Score (APS) III to make the model more robust [3].

### Predicative machine learning models

We ran different machine learning models to predict mortality risks using the structured EHR data. Table 2 shows the performance of various machine learning models; each model presents high sensitivity and specificity. Majority voting and XGBoost performed better than other models. Majority voting had the highest precision, while Gradient boosting had the highest recall. Both Majority voting and XGBoost had the best AUC.

**Fig. 1:**
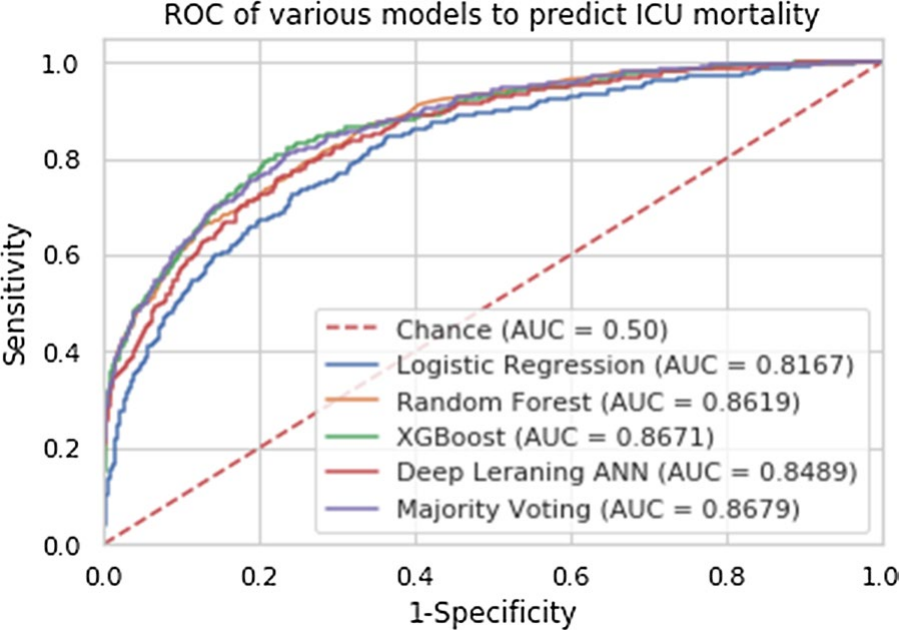
Receiver operating curve (ROC) of machine learning models

Figure 1 presents the ROC for the machine learning models. When we put all the variables of interests into different models, the AUC of Majority Voting was 0.8666, which suggests that the model could predict mortality well.

### Knowledge-guided convolutional neural networks

Table 3 shows the performance of CNN using word embedding and knowledge-guided CNN using CUI + word embedding. We note that the CNN model with word embeddings performed better than the assistant with CUIs, which means adding CUI embeddings as additional input did not improve the performance for this cohort. This is likely due to the features of diabetic diseases, as CUIs were ambiguously connected to their embeddings rather than providing more semantic information. Meanwhile, MetaMap may generate some unnecessary noise, such as irrelevant CUIs [11]. Also, some useful medical concepts may not be recognized, while some false medical concepts may be wrongly recognized when applying MetaMap.

**Table 3:** Performance of CNN using word embedding and knowledge-guided CNN using CUI + word embedding

	AUC	PPV	TPR	F1 score
CNN: text + CUIs	0.88	0.8915	0.9898	0.9381
CNN: text	0.97	0.9587	0.9133	0.9354

Even so, the knowledge-guided CNN model with word embeddings still performed better than machine learning results which just utilized structured EHR data. Further studies are needed, for instance, filtering CUIs based on semantic types may improve the performance.

## Discussion

Chronic diseases introduce multi-factorial issues to patients and healthcare systems, especially to critically ill patients in ICU [34]. This study contributes to different aspects that include the comparison of performance of different data representation and the supervised learning tools, such as machine learning on EHR data and NLP approaches on the medical subdomain classification using the clinical unstructured data. We also concluded that the NLP method using the UMLS concept restricted to semantic information based on the bag-of-concepts feature yielded better optimal results. The use of the standardized terminology proved to be a good knowledge representation approach, thereby leading to the possibility of future clinical EHR system integration. Likewise, the word vectors trained by our datasets may also be useful for future clinical machine learning tasks.

We also propose that our method can be used for clinical notes without medical specialization information. Identifying the clinical subdomain of a clinical note may assist clinicians in mitigating patients’ unsolved problems to adequate medical specialties and experts in time. This algorithm-based method will also assist health care providers to make clinical decisions and provide the best possible care to all the critically ill patients with diabetes.

## Conclusion

In this study, we developed several predictive models to interpret the mortality of diabetes mellitus patients admitted in ICU. We observed the different performance of predictive machine learning models and their interpretability of the NLP models based on the feature sets extracted from the clinical notes. We predicted the mortality of ICU patients, taking into consideration the various factors that had statistically significant impacts on mortality. Based on the results, it is evident that the medical subdomain can be classified accurately using the clinically interpretable supervised learning based on NLP approaches.

We applied rule-based feature engineering and knowledge-guided deep learning approach to train a knowledge-guided CNN model with word embeddings and UMLS CUIs entity embeddings. The evaluation results show that the CNN model is effective for learning hidden features. Although CUI embeddings did not introduce improvement to the whole performance of the NLP model, they were still very helpful when building clinical text representations. More clinical databases and different patient cohorts are needed to evaluate our model in the future.

## Data Availability

The datasets generated and/or analyzed during the current study are available on the MIMIC-III critical care database at https://mimic.physionet.org/. The relevant code and analyses are available at https://github.com/yao8839836/obesity.

## Abbreviations

UMLS: Unified medical language system
NLP: Natural language processing
CNN: Convolutional neural network
CUI: Concept Unique Identifiers
ICU: Intensive care unit
EHR: Electronic health record
SOFA: Sequential organ failure assessment
SAP II: Simplified Acute Physiology Score II
APS III: Acute Physiology Score III
ROC: Receiver operating curve
AUC: Area under the curve
IQR: Interquartile range
LOS: Length of stay
MODS: Multiple organ dysfunction syndrome
PPV: Positive predictive value
TPR: True positive rate
